# Neural correlates of the numerical distance effect in children

**DOI:** 10.3389/fpsyg.2013.00663

**Published:** 2013-10-18

**Authors:** Christophe Mussolin, Marie-Pascale Noël, Mauro Pesenti, Cécile Grandin, Anne G. De Volder

**Affiliations:** ^1^Laboratory Cognition, Language, Development, Center for Research in Cognition and Neurosciences, Université Libre de BruxellesBrussels, Belgium; ^2^Centre de Neurosciences Système et Cognition, Institut de Recherche en Sciences Psychologiques, Université Catholique de LouvainLouvain-la-Neuve, Belgium; ^3^School of Medicine, Institute of Neuroscience, Université Catholique de LouvainBrussels, Belgium; ^4^Pediatric Neurology Service, Cliniques Universitaires St. LucBrussels, Belgium

**Keywords:** number magnitude, children, intraparietal sulcus, frontal cortex, development

## Abstract

In number comparison tasks, the performance is better when the distance between the numbers to compare increases. It has been shown that this so-called numerical distance effect (NDE) decreases with age but the neuroanatomical correlates of these age-related changes are poorly known. Using functional magnetic resonance imaging (fMRI), we recorded the brain activity changes in children aged from 8 to 14 years while they performed a number comparison task on pairs of Arabic digits and a control color comparison task on non-numerical symbols. On the one hand, we observed developmental changes in the recruitment of frontal regions and the left intraparietal sulcus (IPS), with lower activation as age increased. On the other hand, we found that a behavioral index of selective sensitivity to the NDE was positively correlated with higher brain activity in a right lateralized occipito-temporo-parietal network including the IPS. This leads us to propose that the left IPS would be engaged in the refinement of cognitive processes involved in number comparison during development, while the right IPS would underlie the semantic representation of numbers and its activation would be mainly affected by the numerical proximity between them.

## Introduction

Being able to compare two numbers according to their magnitudes is a prerequisite to acquire and master mathematical abilities during childhood. In this kind of task, latencies and error rates decrease as the numerical difference between two numbers to be compared increases (Moyer and Landauer, 1967). The classical interpretation of this so-called numerical distance effect (NDE) is that numbers are automatically converted into an internal representation like analog magnitudes that are in turn compared with each other [but see Verguts and Fias (2004), for a different view]. Since this seminal observation, the NDE has been replicated and manipulated in a large body of adult studies.

Only a few behavioral studies investigate how the NDE evolves during development and the published findings are unclear. Sekuler and Mierkiewicz (1977) assessed performance of children from kindergarten, first-, fourth-, and seventh-grade as well as performance of adult students while they had to compare pairs of Arabic digits. Although the NDE affected performance in all age groups, as reflected by shorter latencies for far distances relative to close distances, the difference in processing speed was more pronounced for younger participants than for older ones. This finding was also reported in other experiments (Duncan and McFarland, 1980; Holloway and Ansari, 2009). The age-related change in the size of NDE could be interpreted as a greater efficiency to access or represent number magnitude as the age increases. Alternatively, it is also possible that the flatter NDE with age corresponds to gradually faster domain-general speed of processing. Indeed, similar developmental changes in the distance effect were observed in number, brightness, and height comparisons (Holloway and Ansari, 2008), suggesting that at least some of the cognitive mechanisms involved in the NDE may be shared by other quantity dimensions.

With the emergence of functional neuroimaging techniques, there were numerous attempts to define which brain areas are responsible for the NDE in adults. Using positron emission tomography (PET) or functional magnetic resonance imaging (fMRI) these experiments provided demonstration that brain areas in and around the intraparietal sulcus (IPS) in both hemispheres were consistently activated during number comparison (Pinel et al., 1999; Pesenti et al., 2000), even in the absence of explicit processing of number magnitude (Eger et al., 2003). Crucially, the extent of brain activation in these cortical areas was found to be modulated by the NDE. Indeed, the brain activation level in the left and right IPS was higher when numerical distance was smaller, both for Arabic numbers and number words (Pinel et al., 2001; Notebaert et al., 2010) as well as for non-symbolic numerosities (i.e., collections of dots, Piazza et al., 2004; Ansari et al., 2006; Cantlon et al., 2006). These findings indicate that areas around the IPS might be the neural substrate of number magnitude representation in adults (Dehaene et al., 2003).

Several neuroimaging studies aimed to examine the developmental course in brain activity changes related to basic numerical abilities such as Arabic number comparison or numerosity habituation. However, current data are mixed. On the one hand, several right-lateralized parietal regions including the IPS were more strongly modulated by NDE in adults than in 10-year-old children during symbolic number comparison. Inversely, the modulation effect in the right precentral and inferior frontal gyri was stronger in children than in adults (Ansari et al., 2005). It is noteworthy that these results were only partially replicated with a non-symbolic comparison task (Ansari and Dhital, 2006). On the other hand, when adults and 4-year-old children were habituated to numerosities varying either on number or element shape, the IPS activation changes linked to number changes overlapped considerably in both groups (Cantlon et al., 2006). This is in accordance with previous observations using evoked related potentials (ERPs) showing that NDE was associated with similar components, localization and timing in 5-year-old children and adults who compared canonical dot patterns or Arabic digits (Temple and Posner, 1998).

Altogether, behavioral data consistently pointed out a weaker sensitivity to NDE for older participants compared to younger—i.e., reflected by a smaller difference between close and far numerical distances. Several neuroimaging studies also pointed out developmental changes related to NDE reflected by a progressive recruitment of parietal regions with age, coupled with a decreasing engagement of frontal regions. However, it is not clear whether these age-related changes correspond to a refinement of the number magnitude representation or rather an improvement in other processes involved in the NDE such as the access to the magnitude from symbolic numbers. To distinguish between these proposals, it is necessary to examine, amongst brain regions modulated by the NDE, which of them are specifically dedicated to number magnitude processing and whether or not the activity in these regions is also modulated by age. Indeed, as pointed out by different fMRI studies in the past (Pinel et al., 2001, 2004; Cohen Kadosh et al., 2005), not only the regions in and around the IPS showed a modulation of their activity by the NDE but also other regions that are not supposed to play a role in the number magnitude representation. To date, no clear explanation has been provided concerning the role played by these regions outside the parietal cortex in the context of number processing.

The present fMRI study aims at addressing these questions by recording neural responses in children aged from 8 to 14 years who were required to compare two Arabic digits separated by either a close or a far distance. This age range was chosen to ensure that children had sufficient knowledge with Arabic digits to compare the magnitude they convey with a high accuracy (Sekuler and Mierkiewicz, 1977; Holloway and Ansari, 2009). In the current study, we paid special attention to include a color comparison task on pairs of non-numerical symbols whose colors were either close (e.g., red vs. pink) or far (e.g., red vs. blue) from each other^1^. It is important to include such kind of control task in order to tap brain activation changes specifically related to number magnitude processing since several adult experiments have pointed out a recruitment of IPS in non-numerical quantity comparison (Pinel et al., 2004; Cohen Kadosh et al., 2005). Accordingly, subtracting the activity related to color distances from the activity related to numerical distances should allow us to point out which brain regions are recruited by the different steps of number comparison (i.e., accessing numerical symbols, mapping these symbols to the number magnitude representation, and activating these magnitudes), while excluding the common cognitive processes engaged in both tasks (e.g., processing visual inputs, performing a comparison and selecting a response). In addition, to disentangle which brain areas, amongst the activated ones, are involved in the NDE (i.e., isolating those whose activation is dedicated to number magnitude processing from those whose activity is not), we also computed a behavioral index of sensitivity to numerical proximity (dRT score) for each child (see Materials and Methods). We used individual (dRT) scores in a correlation analysis to examine the effect of (im)precision of the number magnitude representation on brain activation patterns. By analyzing correlations between brain activity and age or dRT scores, we aimed to get further insight into the cognitive processes engaged in the NDE during development. A common correlation between IPS activity and age and dRT scores (in opposite directions^2^) would indicate that the age-related changes in the size of NDE observed in behavioral studies reflect a refining of the number magnitude representation during development. Alternatively, if the regions modulated by age differ from those modulated by dRT scores, this would lead us to postulate that the behavioral improvement associated with the changes in the size of NDE with age does not tap a refining of the number magnitude representation *per se*, but rather would refer to less specific processes playing a role in the access to this representation or in the comparison between magnitudes.

## Materials and methods

### Participants

Nineteen children aged from 8 to 14 years (6 girls, 2 left-handed, average age 10.5 ± 1.7 years) participated in the fMRI experiment. All participants had normal or corrected-to-normal vision. They were healthy and medication-free with no history of neurological illness or learning disability. All protocols were approved by the local ethics committee of the UCL school of medicine, and have been conducted according to the principles expressed in the Declaration of Helsinki. All legal guardians of the children gave informed written consent prior to the experiment.

### Experimental tasks

During the fMRI sessions, participants performed two tasks each comprising two distance levels, giving rise to four conditions presented in separate blocks. In the number comparison task, two Arabic digits from 1 to 9, separated by close (1 or 2) or far (5 or 6) numerical distances were presented to the participants. In the color comparison task, two non-numerical symbols (selected amongst the symbols Λ, Δ, Ω, Σ, Γ, δ, &, Φ) were presented. The target symbol was red and the color of the other symbol was either close to (i.e., pink) or far from (i.e., blue) red. The pairs of Arabic digits or non-numerical symbols appeared every 1800 ms. Stimuli were flashed for 200 ms at the center of the screen, on both side of a fixation point, followed by a fixed 1600 ms interval. The participants were instructed to keep their eyes on the fixation point throughout the experiment and to avoid movements as much as possible. The participants held a MRI-compatible response button in each hand, and had to select the larger digit (numerical task) or the red symbol (color task) of each pair by pressing the corresponding left or right button as quickly as possible. The position of the correct response was counterbalanced for each trial. Response latencies were measured from the disappearance of the stimuli. During the fixation periods, participants were asked to look at the fixation point without making head or eye movement. Stimuli were presented using a video projector and a translucent screen. The experiment was programmed and responses recorded using E-Prime 1.2 software (Schneider et al., 2002).

Before the scanning session, all participants were carefully instructed about the whole procedure and had to solve four blocks of practice trials outside the MRI room to familiarize with the tasks.

### Neuropsychological tasks

Children's general cognitive abilities were assessed as follows. Intellectual capacities were evaluated using the Similarities and Images Completion subtests of the WISC-III (Wechsler, 1996) which enabled to calculate an estimate of IQ for each child. Four measures of short-term memory were also obtained. In the word span tasks, children were presented with increasingly longer series of words and were asked to repeat them in the actual presentation order (forward word span) or in the reverse order (backward word span). The Corsi block-tapping test (Corsi, 1972) provided a measure of spatial short-term memory. In this task, children were asked to reproduce the same sequence of block pointing as shown by the examiner. The listening span [adapted from Daneman and Carpenter (1980)] was used to evaluate the central executive component of the working memory. In this test, the experimenter read sets of sentences (from two to four) and the child was required to indicate whether each sentence was true or not. Then, at the end of the set, the child had to recall the last word of each of the sentences included in the set. Individual scores in these neuropsychological tasks were in the normal range. A summary of individual results is provided in Supplementary Table S1.

### Behavioral data analysis

An individual measure of the sensitivity to numerical proximity was obtained following an approach adapted from that of Holloway and Ansari (2009). For each child, reaction times (RTs) for comparisons with far (median of distances 5 and 6) distances were subtracted from those with close (median of distances 1 and 2) numerical (or color) distances. These values were then divided by the RTs for far distance comparisons to obtain a normalized score for each participant controlled for the differences in speed of processing. A similar method was used to compute the individual sensitivity to color proximity. Since some of the processing stages are common in both numerical and color comparison tasks, we computed the difference between the two RT measures by subtracting the index of sensitivity to color proximity from the index of sensitivity to numerical proximity. The resulting score (dRT) corresponds to a measure of (im)precision of the number magnitude representation, excluding cognitive mechanisms that are not specific to number processing. The higher the dRT score the larger is the overlap between the number magnitudes, and the lower is the precision in comparing symbolic numbers. The individual dRT scores were then entered in regression analyses to examine their influence on task-related brain activity changes.

### fMRI acquisition

Blood oxygen level-dependent (BOLD) functional images were acquired in a 1.5 T MRI unit (Gyroscan, Philips Medical Systems), using a multislice T2^*^-weighted gradient echo-planar imaging (EPI) sequence [TR (repetition time), 3000 ms; TE (echo time), 50 ms; flip angle, 90°] with 33 axial slices, 3.6 mm slice thickness (isotropic voxel), in the bicommissural orientation. The matrix was 64 × 64 and the field of view was 210 × 210 mm. Structural high-resolution T1-weighted 3D gradient echo images (Fast field echo, TR, 30 ms; TE, 3 ms; flip angle, 30°; slice thickness, 1.5 mm) were also acquired.

The stimuli were backprojected using a MRI compatible projector placed at the rear of the magnet and viewed through a tilted mirror mounted on the head coil (Silent Vision® System, Avotec, Inc., http://www.avotec.org). Foam pads were used to restrict head movements. The fMRI paradigm consisted of three runs of eight alternating epochs of comparison tasks (36 s per epoch) and fixation periods (18 s). Each run comprised the acquisition of 144 volumes and contained 160 trials (20 trials × 4 conditions × 2 blocks of each condition per run). Stimulus onset was synchronized with the acquisition of the first slice. The participants received instructions before each sequence, and were not warned of the alternation between tasks and conditions. In each run, two number comparison tasks (close distances and far distances) and two color comparison tasks (close colors and far colors) were presented in pseudo-random order.

### fMRI data analysis

Functional data processing and statistical analyses were carried out using Statistical Parametric Mapping (SPM 2, The Wellcome Department of Imaging Neuroscience, London, UK, http://www.fil.ion.ac.uk/spm), implemented in Matlab (Mathworks Inc., Sherborn MA, USA). The first six volumes of each run were discarded to allow for T1 equilibration. All individual images were than realigned to the first remaining fMRI volume of the corresponding participant to correct for within- and between-run motion, coregistered with the individual anatomical scan, and further spatially normalized using the adult MRI template supplied by the Montreal Neurological Institute (MNI). This procedure resulted in normalized fMRI images with a cubic voxel size (2 × 2 × 2 mm). Next, a spatial smoothing with a Gaussian kernel of 8 mm (full width at half maximum, FWHM) was applied in order to reduce the residual anatomical and functional variability across participants. The means (SD) of head movements of the children in the *x, y*, and *z* plane were 0.1 (0.3), 0.6 (0.8), 1.3 (1.4) mm, respectively.

Condition-related changes in regional brain activity were estimated for each participant by a general linear model (GLM) in which the responses evoked by each condition of interest were modeled by a standard hemodynamic response function. The contrasts of interest were first computed at the individual level to identify the cerebral regions significantly activated by numerical (close numerical distance vs. far numerical distance) and color (close color distance vs. far color distance) distances, each condition relative to the fixation periods used as a general baseline. Brain activation maps for the critical contrast (close numerical distance vs. far numerical distance) − (close color distance vs. far color distance) [for a detailed method, see Henson and Penny (2003)] were then entered into a group-level random-effect analysis using a GLM with either age or dRT scores as the covariates of interest. Significant voxels clusters of activation were identified using a threshold of *P* < 0.001 (uncorrected) and an extent threshold of *P* < 0.05, corrected for multiple comparisons, at the cluster level (less than 0.05 under the false discovery rate at the voxel level; see Genovese et al., 2002). The foci that were significantly activated at a corrected *P* < 0.05 (cluster level) or a corrected *P* < 0.05 (FDR, voxel level) were considered. Next, in all brain areas found with RFX analysis, correlation analyses were performed between the beta weights of the contrast and the age or the dRT scores, in order to find out the key area(s) modulated either by age or by the sensitivity to NDE. In this correlation analysis, we applied a cluster size threshold to each significant region resulting from the group-level random-effect analysis using a sphere of 10 mm around the peak of activation. Then, the individual degree of activity (beta values) for each region was correlated with age and dRT score. Anatomical labels were given on the basis of the classification of the AAL (automated anatomical labeling) atlas (Tzourio-Mazoyer et al., 2002).

## Results

### Behavioral data

Performance in the two tasks was very accurate (95 and 98% of correct responses in numerical and color tasks, respectively) and fast (numerical task: 620 ms; color task: 505 ms). An ANOVA was conducted on median RTs of each participant with task (numerical or color comparisons), and distance (close or far) as within-subject factors. The effect of task was significant [*F*_(1, 18)_ = 61.79, η_partial_ = 0.78, *P* < 0.001], indicating that the number comparison task was more time-demanding than the color comparison one. As expected, the far distances yielded faster RTs than close ones [*F*_(1, 18)_ = 85.53, η_partial_ = 0.83, *P* < 0.001] in both tasks [numerical task: *F*_(1, 18)_ = 91.36, η_partial_ = 0.84, *P* < 0.001, 567 and 673 ms; color task: *F*_(1, 18)_ = 5.61, η_partial_ = 0.25, *P* = 0.03, 494 and 516 ms] although the impact of this factor was stronger in numerical than color comparisons as indicated by the Task × Distance interaction [*F*_(1, 18)_ = 32.49, η_partial_ = 0.66, *P* < 0.001].

### Relation between age and dRT scores

In line with previous data (Holloway and Ansari, 2009; Mundy and Gilmore, 2009), our measure of individual sensitivity to numerical proximity (dRT) varied from −0.01 to 0.54 (*M* = 0.26; *SD* = 0.17) and showed a negative correlation with age (*r* = − 0.49, *P* < 0.05), reflecting a decreasing receptivity to the NDE during development. Moreover, no correlation was found between dRT scores and any neuropsychological measure (all *P*s > 0.05).

### Relation between age and brain activation

**Figure 1:**
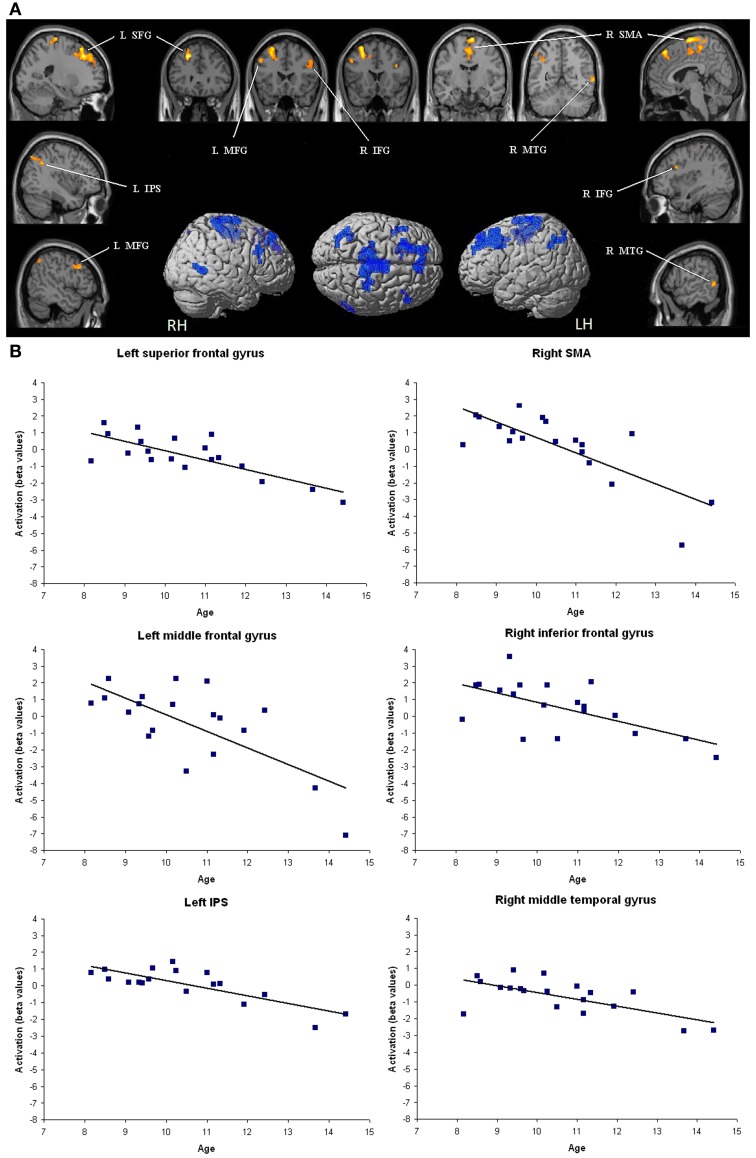
**Brain areas that showed significant decreases in activation with age. (A)** Brain regions (*P* < 0.05 corrected for multiple comparisons at the cluster level) are superimposed on sagittal and coronal sections of an individual normalized brain MRI. Surface rendering of significant areas of activation that are negatively correlated with age are superimposed on a standard MRI brain for reference. IFG, inferior frontal gyrus; IPS, intraparietal sulcus; MFG, middle frontal gyrus; MTG, middle temporal gyrus; SFG, superior frontal gyrus; SMA, supplementary motor area. **(B)** Bar charts depict decreases in activation (individual beta values) with age in each relevant brain area across children.

As illustrated in Figure 1, amongst brain regions activated in the contrast [(close – far numerical distance) – (close – far color distance)], significant negative correlations with age emerged in the left superior frontal gyrus (*r* = 0.77, *P* < 0.001), middle frontal gyrus (*r* = −0.71, *P* < 0.01), and IPS (*r* = −0.79, *P* < 0.001). Right lateralized activation foci that decreased with age were also found in the SMA (*r* = −0.78, *P* < 0.001), inferior frontal gyrus (*r* = −0.62, *P* < 0.01), and middle temporal gyrus (*r* = −0.67, *P* < 0.01). Children did not show increase in brain activation with age (whole brain analysis). The impact of age on brain activation in all these brain areas remained significant even when dRT scores were partialled out (see Table 1).

**Table 1:** **Brain areas that showed significant negative correlations with age**.

**Brain region**	**No. of voxels**	***Z*_max_**	**Talairach coordinates**			**Correlation**	
			***x***	***y***	***z***	***r***	***r*_partial_**
Left superior frontal gyrus	1181	5.38	−22	30	44	−0.77^**^	−0.83^**^
Left middle frontal gyrus	119	3.70	−48	22	36	−0.71^*^	−0.61^*^
Left IPS	229	4.00	−38	−52	36	−0.79^**^	−0.77^**^
Right SMA	2012	4.94	6	−10	74	−0.78^**^	−0.79^**^
Right inferior frontal gyrus	127	3.82	38	18	28	−0.62^*^	−0.62^*^
Right middle temporal gyrus	115	4.81	66	−50	4	−0.67^*^	−0.80^**^

Brain regions corresponded to activation peaks, obtained from random-effect analysis using a threshold of P < 0.001, uncorrected, at the voxel level and P < 0.05, corrected for multiple comparisons, at the cluster level. Both simple negative correlations (r) and partial negative correlations (r partial, i.e., corrected for variance explained by dRT scores) are reported. Coordinates are reported in MNI space as given by SPM2 and correspond only approximately to Talairach and Tournoux space (Talairach and Tournoux, 1988). Anatomical labels are based on the AAL (automated anatomical labeling) atlas (Tzourio-Mazoyer et al., 2002).

* *P < 0.01*,

** *P < 0.001*.

### Relation between dRT scores and brain activation

**Figure 2:**
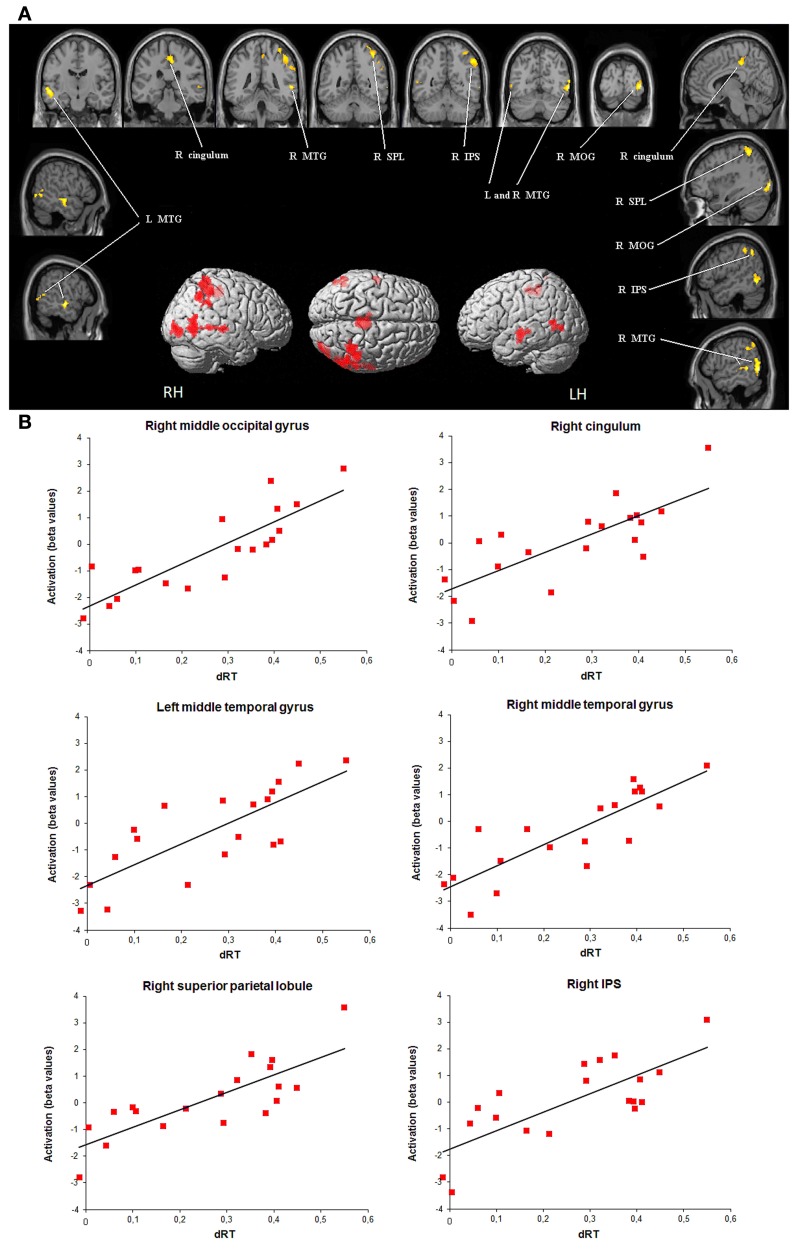
**Brain areas that showed significant increases in activation with sensitivity to numerical distance effect (dRT). (A)** Brain regions (*P* < 0.05 corrected for multiple comparisons at the cluster level) are superimposed on sagittal and coronal sections of an individual normalized brain MRI. Surface rendering of significant areas of activation that are positively correlated with dRT scores are superimposed on a standard MRI brain for reference. IPS, intraparietal sulcus; MOG, middle occipital gyrus; MTG, middle temporal gyrus; SPL, superior parietal lobule. **(B)** Bar charts depict increases in activation (individual beta values) with dRT scores in each relevant brain area across children. dRT score [adapted from Holloway and Ansari (2009)], individual behavioral index of selective sensitivity to numerical proximity (see text).

Children showed only positive correlations between brain activation level and dRT scores used as a measure of sensitivity to the NDE. As illustrated in Figure 2, amongst brain regions activated in the contrast [(close - far numerical distance) - (close - far color distance)], the brain activation level was modulated by dRT scores in the middle temporal gyrus in both hemispheres (left: *r* = 0.78, *P* < 0.001; right: *r* = 0.84, *P* < 0.001). In the right brain hemisphere, a positive correlation with dRT scores was observed in the superior parietal lobule (*r* = 0.80, *P* < 0.001), in and around the IPS (*r* = 0.76, *P* < 0.001), in the middle occipital gyrus (*r* = 0.85, *P* < 0.001), and in the cingulum (*r* = 0.77, *P* < 0.001). In all the above regions, similar results were obtained when the effect of age was partialled out^3^.

We also controlled for the lack of correlation between brain activity level in all significant regions and various individual measures of working memory (forward and backward visuospatial, verbal, and listening spans) and processing speed, such as mean RTs for numerical and color comparison tasks respectively, or mean RTs across both tasks. None of working memory or processing speed measurements correlated with the brain activity in any of the aforementioned brain regions (*P*s > 0.1). Furthermore, the correlations with dRT scores remained significant even after controlling for these factors (Table 2).

**Table 2:** **Brain areas that showed significant positive correlations with individual behavioral indexes of selective susceptibility to numerical proximity (dRT scores)**.

**Brain region**	**No. of voxels**	***Z*_max_**	**Talairach coordinates**			**Correlation**			
			***x***	***y***	***z***	***r***	***r*_partial_**		
							**Age**	**WM^1^**	**Speed^2^**
Left middle temporal gyrus	214	4.41	−56	−14	−8	0.78^**^	0.73^*^	0.73^*^	0.74^*^
Left middle temporal gyrus	114	3.66	−52	−70	0	0.74^**^	0.66^*^	0.68^*^	0.70^*^
Right middle temporal gyrus	291	4.50	58	−66	0	0.84^**^	0.78^**^	0.77^*^	0.88^**^
Right middle temporal gyrus	133	4.37	60	−42	0	0.81^**^	0.75^**^	0.83^**^	0.80^**^
Right superior parietal lobule	352	4.11	38	−50	58	0.80^**^	0.76^**^	0.76^*^	0.80^**^
Right lateral bank of IPS—IPS	176	3.84	52	−54	46	0.76^**^	0.67^*^	0.77^*^	0.71^*^
Right middle occipital gyrus	201	4.79	38	−90	0	0.85^**^	0.85^**^	0.82^**^	0.84^**^
Right middle cingulum	335	3.90	8	−30	44	0.77^**^	0.65^*^	0.70^*^	0.71^*^

Brain regions corresponded to activation peaks, obtained from random-effect analysis using a threshold of P < 0.001, uncorrected, at the voxel level and P < 0.05, corrected for multiple comparisons, at the cluster level. Both simple positive correlations (r) and partial positive correlations (r partial, i.e., corrected for variance explained by age, working memory, and processing speed measures) are reported. Coordinates are reported in MNI space as given by SPM2 and correspond only approximately to Talairach and Tournoux space (Talairach and Tournoux, 1988). Anatomical labels are based on the AAL (automated anatomical labeling) atlas (Tzourio-Mazoyer et al., 2002).

* *P < 0.01*,

** P < 0.001.

1 Working memory measures (forward and backward visuospatial, verbal, and listening spans).

2 Processing speed measures (mean RTs for numerical or color comparisons, mean RTs across tasks).

## Discussion

The present paper investigated changes in neural activity underlying performance during symbolic number comparison in children aged 8–14 years. Our results provide pieces of evidence that brain regions showing activity modulation with age were not the same as those affected by the NDE. Here under, the potential role played by each of the regions whose activity was modulated by age or by selective sensitivity to numerical proximity are discussed with regards to past literature.

Amongst areas that showed higher activation for close relative to far numerical distances, significant negative correlation with age were found in many frontal regions, including the left middle and superior frontal gyri as well as the right supplementary motor area. This means that, compared to young children, the difference of brain activation between close and far numerical distances in the above areas was weaker in older children. These findings are in line with previous data, which indicated a progressive disengagement of frontal brain regions in symbolic number comparison (Ansari et al., 2005) and calculation (Rivera et al., 2005) with age. Importantly, the difference of brain activation between close and far numerical distances decreased with age not only in frontal regions but also in the left IPS. It is tempting to link these age-related neural changes with the behavioral observations here and elsewhere according to which the size of the NDE decreases during development (Sekuler and Mierkiewicz, 1977; Duncan and McFarland, 1980; Holloway and Ansari, 2009). The question then arises what are the roles of these frontal regions and the left IPS in the NDE. It is worth noting that the brain activity level in these brain regions was not modulated by dRT scores used as individual behavioral indices of selective sensitivity to numerical proximity, which could be considered as indicating imprecision in the number magnitude representation. One could therefore postulate that the frontal brain areas and the left IPS were not dedicated to the number magnitude representation *per se*, but rather were engaged in some cognitive processes related to symbolic number processing, such as the connexion between Arabic digits and the related number magnitudes. As age and experience with symbolic numbers increase, these mechanisms become more and more automatic and require lesser resources, which could be reflected by a weaker engagement of the frontal brain areas and the left IPS during development.

Beyond age-related changes, children showed positive correlations between brain activity level in a series of regions and dRT scores. In several brain areas in the right hemisphere including the middle occipital gyrus, the middle temporal gyrus, the IPS, and the superior parietal lobule, the brain activity level was high in children who were particularly influenced by the numerical proximity between Arabic digits, whereas it was lower in children who were less affected by NDE. This pattern of results is observed not only with dRT scores but also with a “pure” measure of NDE as typically computed in previous studies (Holloway and Ansari, 2009; Mundy and Gilmore, 2009). In our opinion, this demonstrate that the brain regions resulting from our critical contrast are engaged in the manifestation of the NDE, and, more particularly, in number magnitude processing. The role of this right-lateralized occipito-temporo-parietal network has been previously interpreted as reflecting the successive steps of cognitive processes engaged in Arabic number comparison. First, the right middle occipital gyrus and the fusiform gyrus are part of a ventral occipito-temporal pathway specialized for the visual recognition of digits (Cohen and Dehaene, 1996; Pesenti et al., 2000). Second, the IPS, especially in the right hemisphere, is systematically activated when numbers are manipulated, whatever the task and independently of number format (Piazza et al., 2007) or notation (Pinel et al., 2001). In adults, its bilateral recruitment was found to decrease quasi-monotonically as the numerical distance increased, in tight parallel with the behavioral performance (Pinel et al., 2001). The same pattern of change in right IPS activity was also observed in 10-year-old children, albeit to a lesser degree (Ansari et al., 2005). Our data confirm and extend these findings by showing that the brain activity in and around the IPS but also in occipital and temporal regions was modulated by the individual response to numerical proximity between the digits to compare. This indicates some activation of number magnitude in the early stages of number processing. This view is in line with the remarkable demonstration produced by Burr and Ross (2008) who showed that a perceived numerosity is susceptible to adaptation, just like the primary visual properties of a scene, e.g., location, color, or physical size of the stimuli. After a simple 30 s adaptation to a patch containing a large number of spots, an identical following patch seems to have fewer elements. According to the authors, the neurons in the IPS are likely the candidates for the neural substrate of this visual “sense of number.” However, as pointed out by Butterworth (2008), earlier neural stages in visual processing could also be involved in the adaptation phenomenon reported by Burr and Ross (2008). This could explain why dRT scores as indices of imprecision in the number magnitude representation also influenced the brain activity in the occipital cortex in the present study.

The present findings favor an involvement of left and right parietal brain areas in number comparison and shed new insight on the specific roles of these brain regions. Whereas the Triple-code model both in its original (Dehaene and Cohen, 1995, 1997) and more recent versions (Dehaene et al., 2003) postulates that left and right IPS areas code the quantity meaning of numbers, past and current research including the present study indicate that the lateralization of number magnitude representation is perhaps more complex. The present observations suggest that the left and right IPS differently contribute to the development of number magnitude processing. The progressive disengagement of regions in the left IPS with increasing age would be related to the refinement of cognitive processes involved but not directly related to number magnitude processing. In contrast, the right IPS would underlie the semantic representation of numbers and its activation would be especially affected by the individual sensitivity to numerical distances between them. This hypothesis accords well with previous reports of a right-hemispheric advantage during number comparison (Dehaene et al., 1996; Chochon et al., 1999; Pinel et al., 2001) as well as other tasks requiring an abstraction of numerical information (Rosselli and Ardila, 1989; Langdon and Warrington, 1997). More direct evidence favoring a right-lateralized representation of number magnitude was provided by Piazza et al. (2007). Using an adaptation paradigm, the authors reported recovery in the right parietal cortex recruitment when Arabic digits or dot patterns changed, and they concluded that this brain area could share neural populations encoding both non-symbolic and symbolic numbers.

In conclusion, the present data extend previous observations by showing that the left and the right IPS as well as other brain regions could contribute differently to number magnitude processing during child development. Further work is clearly needed, however, to determine the precise role of these brain areas in the acquisition of elaborate numerical and non-numerical knowledge.

## Author contributions

Conceived and designed the experiments: Christophe Mussolin, Marie-Pascale Noël, Mauro Pesenti, Cécile Grandin, and Anne G. De Volder. Performed the experiments: Christophe Mussolin and Anne G. De Volder. Analyzed the data: Christophe Mussolin and Anne G. De Volder. Wrote the paper: Christophe Mussolin, Anne G. De Volder, and Mauro Pesenti.

### Conflict of interest statement

The authors declare that the research was conducted in the absence of any commercial or financial relationships that could be construed as a potential conflict of interest.

## Footnotes

^1^In our control task we have chosen to use non-numerical symbols and to avoid colored Arabic digits in order to prevent automatic activation of number magnitude related to them (Girelli et al., 2000; Rubinsten et al., 2002), which could in turn lead to brain activation around the IPS (Pinel et al., 2004).

^2^In this case, positive correlations between dRT and IPS activity are expected since a high sensitivity to numerical proximity should be associated with a larger activation in brain regions dedicated to number magnitude processing. By contrast, IPS regions should be negatively correlated with age since a lesser requirement of these regions is expected as experience with number magnitudes increases.

^3^We also analyzed the correlations between the level of activity in areas resulting from the critical contrast (close numerical vs. far numerical) – (close color vs. far color) and the sensitivity to NDE as typically computed in previous studies (Holloway and Ansari, 2009; Mundy and Gilmore, 2009). It appeared that the activity in all regions positively correlated with this “pure” measure of numerical distance effect even when controlled for age (*r* from 0.23 to 0.70) but not with the similar measure of color distance effect (*r* from −0.69 to −0.17).
