# Supplementary material for: Neural correlates of the numerical distance effect in children
Source: Front Psychol. 2013 Oct 18;4:663. doi: 10.3389/fpsyg.2013.00663 (PMC3798761; doi:10.3389/fpsyg.2013.00663)
Supplement: Supplementary file 1 [file DataSheet1.DOC]

Table S1. Descriptive information and mean performance

|  | **Children** | **Normative data** |
| --- | --- | --- |
|  | *Mean (SD)* | *Mean (SD)* |
| *Descriptive information* |  |  |
| *N* | 19 |  |
| Gender (M/F) | 13/6 |  |
| Age (in month) | 10.5 |  |
|  |  |  |
| *Working memory* |  |  |
| Verbal span (forward) | 3.9 (0.6) | 3.5 (0.5) |
| Verbal span (backward) | 3.2 (0.4) | - |
| Visuo-spatial span | 5.5 (0.9) | 5.3 (0.9) |
| Listening span | 3.3 (0.8) | 2.2 (0.4) |
|  |  |  |
| *IQ* |  |  |
| Image completion (raw scores) | 11.8 (2.4) | 9.8 (3.2) |
| Similarities (raw scores) | 13.3 (2.4) | 10.1 (2.6) |
| Estimated IQ | 114.8 (11.5) | - |
|  |  |  |
| *Reading* |  |  |
| LUM (correct responses) | 75.7 (22) | 72 (16.1) |
| L3 (correct responses) | 25.7 (8.7) | 23.1 (6.4) |

*Note*. Standard deviations are shown in parentheses.
